# UDDIPOK: A reading comprehension based question answering dataset in Bangla language

**DOI:** 10.1016/j.dib.2023.108933

**Published:** 2023-02-02

**Authors:** Tanjim Taharat Aurpa, Md Shoaib Ahmed, Richita Khandakar Rifat, Md. Musfique Anwar, A.B.M. Shawkat Ali

**Affiliations:** a Department of Computer Science and Engineering, Jahangirnagar University, Savar, Dhaka, Bangladesh; b Department of Computer Science and Engineering, International University of Business Agriculture and Technology, Bangladesh; c Department of Information and Computer Science, King Fahd University of Petroleum and Minerals, Saudi Arabia; d Central Queensland University, Melbourne, Australia; e Brian Station 23 Ltd, Dhaka, Bangladesh; f JU Data Mining Research Lab, Dhaka, Bangladesh

**Keywords:** Bangla NLP, Bangla reading comprehension, Bangla question answering, Reading comprehension, Reading comprehension based on QA, Bangla reading comprehension dataset, Question answering Bangla dataset

## Abstract

The popularity of reading comprehension (RC) is increasing day-to-day in Bangla Natural Language Processing (NLP) research area, both in machine learning and deep learning techniques. However, there is no original dataset from various sources in the Bangla language except translated from foreign RC datasets, which contain abnormalities and mismatched translated data. In his paper, we present UDDIPOK, a novel wide-ranging, open-domain Bangla reading comprehension dataset. This dataset contains 270 reading passages, 3636 questions, and answers from diverse origins, for instance, textbooks, exam questions from middle and high schools, newspapers, etc. Furthermore, this dataset is formated in CSV, which contains three columns: passages, questions, and answers. As a result, data can be handled expeditiously and easily for any machine learning research.

**Specification Table**

**Table:** 

Subject	Bangla Natural Language Processing, Automated Education System
Specific Subject Area	Reading Comprehension-based Question Answering in the Bengali Language
Type of Data	Text/String
Data Format	Raw
Description of Data Collection	This data is generated by collecting real-world passages. We created questions from those passages and annotated the answers. The main motive was to develop a real-time dataset that could be used in the education system.
Data Source Location	Jahangirnagar University, Zone: Savar, Dhaka Country: Bangladesh
Data Accessibility	Repository name: Mendeley Data Data identification number (DOI number): Link of the dataset: https://data.mendeley.com/datasets/s9pb3h2cjy/3
Related Research	[1] Aurpa, T. T., Rifat, R. K., Ahmed, M. S., Anwar, M. M., & Ali, A. S. (2022). Reading comprehension based question answering system in Bangla language with transformer-based learning. Heliyon, 8(10), e11052. The progress of Natural Language Processing (NLP) is not significant in Bangla Language. To bring this progress introducing new datasets and research methodology should be emphasized. Therefore we have created a new Bangla RC-based dataset ‘UDDIPOK'. The main objective of this dataset is to contribute to Reading Comprehension (RC) based question-answering systems. The dataset is created from real-world Bangla content, which can help to develop practical systems in Bangla education. Authors from [1] have used this dataset to develop an RC system in Bangla Language using transformer-based architecture. A large portion of this dataset is used to train the models and the remaining portion is utilized to test the performance of the trained model.

**Value of the Data**

• During COVID-19, all education systems focused on online/virtual solutions. Various data related to educational patterns are required to make automated solutions. This dataset can be used to create automated reading comprehension (RC) systems.
• The world's sixth-largest language is Bengali. Almost 228.7 million people from Bangladesh and India speak it as their first language. The historical interest in this language is so great that UNESCO honored the Bangla language martyrs by declaring 21st February as International Mother Language day. Still, the research in the Bangla language gets significantly less attention. Therefore, the development of these datasets can enrich Bangla NLP.
• A large number of students in Bangladesh are studying in Bangla medium and need different automated systems in the Bangla language. This dataset can help the research on Bangla educational systems.
• Existing data for RC is generated by translating from English datasets. This dataset is collected from Bangla articles, biography, fiction, etc. Thus, this real-time dataset can assemble practical solutions. Therefore passively, it can contribute to modern Bangla education system.
• The contexts in the existing datasets are very simple and short, e.g., one lined context for one question. Unlike existing datasets, UDDIPOK contains significant long passages and questions, which increase its real-time value for training deep learning models

## 1 Objective

The progress of Natural Language Processing (NLP) is not significant in Bangla Language. To bring this progress introducing new datasets and research methodology should be emphasized. Therefore, we have created a new Bangla RC-based dataset ‘UDDIPOK'. The main objective of this dataset is to contribute to Reading Comprehension (RC) based question-answering systems. The dataset is created from real-world Bangla content, which can help to develop practical systems in Bangla education.

Authors from [1] have used this dataset to develop an RC system in Bangla Language using transformer-based architecture. A large portion of this dataset is used to train the models and the remaining portion is utilized to test the performance of the trained model.

## 2 Data Description

With the soaring demand for online education systems, RC-based question answering systems are gaining tremendous popularity and research attention. Numerous language-centric research on RC is conducted day by day. However, the Bangla NLP research is diminishing in this race. By perceiving this urgency in the Bangla language, we developed a real-time dataset named ‘UDDIPOK.’ The word ‘UDDIPOK’ is a Bangla word, and it means ‘Stimulus.’ In Bangla RC, the given passage is called ‘UDDIPOK,’ which students follow to answer the questions. For this motivation, we named our dataset ‘UDDIPOK.’

The dataset contains two files, and the file description will be discussed here. One file is the actual data which is in Bangla language. This file has Bangla passages, questions, and answers. The passages, questions, and answers in UDDIPOK have different lengths. The average length (Average number of characters) of the passages, questions and answers are 379, 83, and 1, respectively. We also figured out the maximum and minimum word count of the dataset UDDIPOK. The maximum characters for passages, questions, and answers are 822, 317, and 42, and the minimum is respectively 61, 5, and 2. Besides character counts, we identify the word counts also. All of these pieces of information are mentioned in Table 1. These pieces of information are determined from raw data.

**Table 1:** UDDIPOK in numbers (Here we mentioned all numeric information, such as Total count, Maximum, Minimum, and Average about our dataset).

	Total	Maximum Word count	Maximum Character count	Minimum Word count	Minimum Character count	Average Numbers of Words	Average Numbers of characters
Passage	270	133	822	10	61	57	379
Question	3636	53	317	2	5	13	83
Answer	3636	7	42	1	2	1	8

Another file is the English translation of the Bangla data. We use Google Translate (https://translate.google.com/) for the translation task of our dataset.

The UDDIPOK dataset is created to train models developed for generating answers for given input passages and questions. There are 3636 observations in the dataset, each containing a passage (context), corresponding questions, and answers. The passages are collected from different Bengali articles, fiction, biographies, etc. After that, the questions and answers are annotated carefully, considering real-world questions and answers. A glimpse of UDDIPOK with English translation has been shown in Table 2.

**Table 2:** Sample reading comprehension (passages, questions, and answers) of the UDDIPOK dataset and the english translations.

In the next section, we have mentioned the experimental works of this dataset.

## 3 Experiment on Data, Materials and Methods

### 3.1 Experimental Environment

For data collection, we utilized the Google cloud-based form called google sheet and stored it in CSV format. The local machine used for this data collection process contains AMD Ryzen 7 5700U CPU and 16GB RAM. For training the deep learning models, we use Google Colab, Google's cloud-based notebook. It provides GPU and TPU and is executable with Ubuntu OS and Tesla k-80 GPU of NVIDIA along with 2 GB of GPU memory.

### 3.2 Data Preprocessing

Before using the data for any downstream tasks, the data need to be clean. Otherwise, it may perform poorly, as the raw text has unnecessary characters, stop words, etc. The following preprocessing steps [2] succor in increasing accuracy for classifiers:

• Removal of punctuation marks (’.’, ’?’, ’|’, etc.), special characters (’#’, ’$’, ’&’, etc.) etc. helps in the high performance in downstream task. We have removed these unnecessary characters from the data.
• Bangla stop words such **,** etc. have no significance in the deep learning tasks. Therefore removing these stop words is vital before using this dataset.
• Finally, we applied lemmatization and stemming on the text for determining the roots of words. For example,  is the root word for , , etc. So the determination of the root word or lemma can be helpful for downstream tasks.

**Fig. 1:**
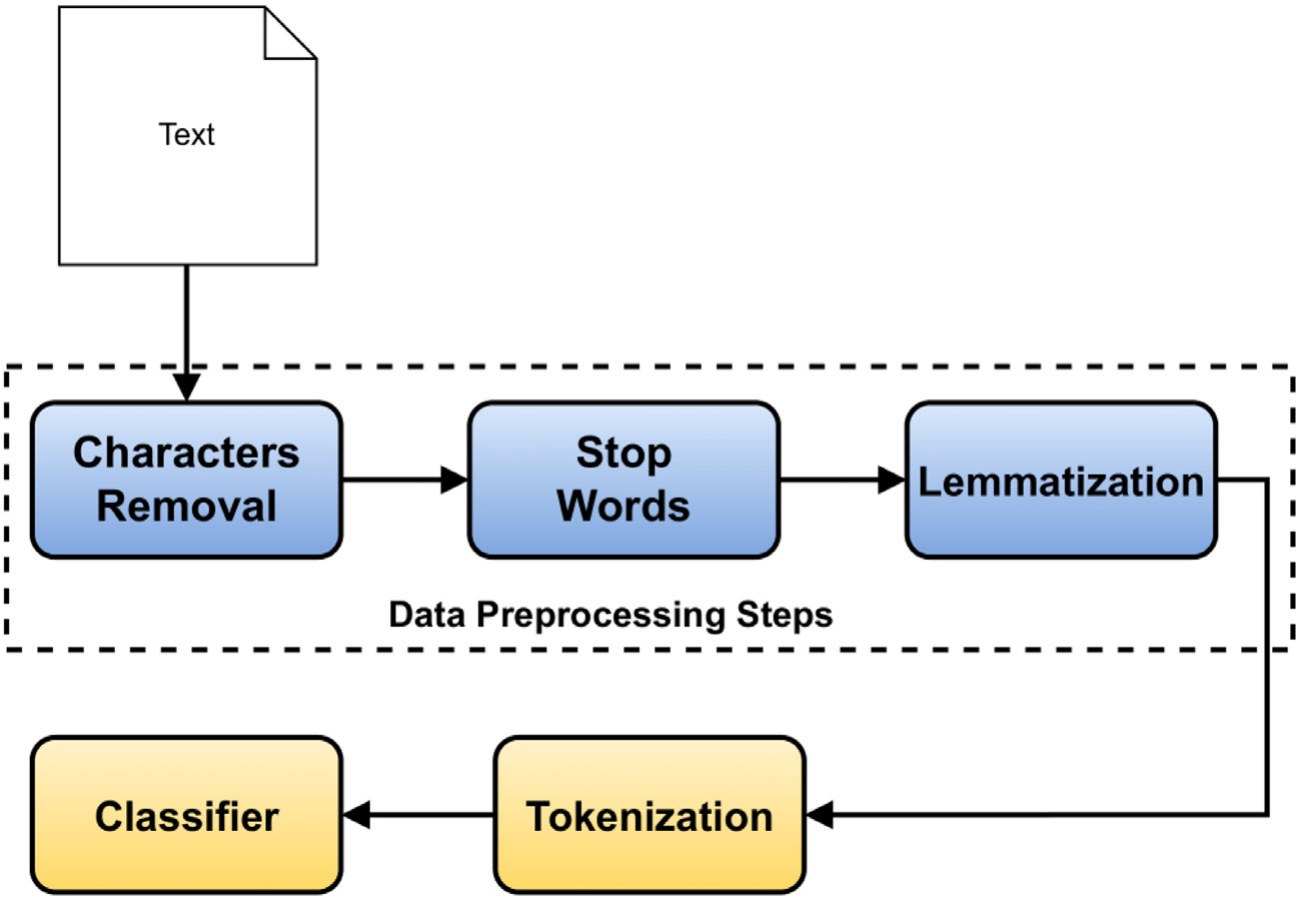
The preprocessing steps of the UDDIPOK dataset before applying to models.

The preprocessing steps for any regression or deep learning problem is sketched out in Fig. 1.

### 3.3 Data Validation

To validate our dataset, we applied the data to remarkable NLP architectures. At first, we trained recurrent deep learning models such as Long Short Term Memory (LSTM), Bi-LSTM with attention, and Simple Recurrent Neural Networks (RNN) with our dataset and obtained satisfactory performance with these models. Transformer-based architectures provide better results than other models. Therefore we applied the dataset to transformer-based architectures BERT (Bidirectional Encoder Representations from Transformers) [4] and ELECTRA (Efficiently Learning an Encoder that Classifies Token Replacements Accurately) [3].

The performance of these models on our data is significant. The lowest accuracy is 73.23%. Among all models, the BERT architecture provides the highest accuracy, 87.78%. We also determine the F1 scores for these classifiers. The accuracy and F1 Score of all these models are represented in Table 3.

**Table 3:** Comparing classifiers with their accuracy and F1 scores (a comparison of the accuracy and F1 scores of several deep neural network architectures has been mentioned here).

Classifier	Accuracy	F1 Score
BERT	87.78	93.49
ELECTRA	82.52	90.42
Bi-LSTM with Attention	80.09	87.90
LSTM	77.41	82.88
Simple RNN	73.23	79.01

## Ethics Statements

The authors of this work have read and agree with the ethical requirements.

## CRediT authorship contribution statement

**Tanjim Taharat Aurpa:** Data curation, Validation, Writing – original draft. **Md Shoaib Ahmed:** Conceptualization, Investigation. **Richita Khandakar Rifat:** Data curation, Validation. **Md. Musfique Anwar:** Supervision. **A.B.M. Shawkat Ali:** Supervision.

## Declaration of Competing Interest

We authors declare that we have conducted this work without the influence of any competing financial benefits and personal relationships.

## Data Availability

UDDIPOK: Reading Comprehension Based Question Answering Dataset in Bangla Language (Original data) (Mendeley Data).
